# Location analysis of presynaptically active and silent synapses in single-cultured hippocampal neurons

**DOI:** 10.3389/fncir.2024.1358570

**Published:** 2024-04-23

**Authors:** Otoya Kitaoka, Kohei Oyabu, Kaori Kubota, Takuya Watanabe, Satoru Kondo, Teppei Matsui, Shutaro Katsurabayashi, Katsunori Iwasaki

**Affiliations:** ^1^Department of Neuropharmacology, Faculty of Pharmaceutical Sciences, Fukuoka University, Fukuoka, Japan; ^2^Department of Physiology, School of Medicine, The University of Tokyo, Tokyo, Japan; ^3^World Premier International Research Center-International Research Center for Neurointelligence (WPI-IRCN), The University of Tokyo Institutes for Advanced Study (UTIAS), Tokyo, Japan; ^4^Laboratory of Neurocomputation, Graduate School of Brain Science, Doshisha University, Kyoto, Japan

**Keywords:** silent synapse, single neuron, Sholl analysis, autapse culture, location analysis

## Abstract

A morphologically present but non-functioning synapse is termed a silent synapse. Silent synapses are categorized into “postsynaptically silent synapses,” where AMPA receptors are either absent or non-functional, and “presynaptically silent synapses,” where neurotransmitters cannot be released from nerve terminals. The presence of presynaptically silent synapses remains enigmatic, and their physiological significance is highly intriguing. In this study, we examined the distribution and developmental changes of presynaptically active and silent synapses in individual neurons. Our findings show a gradual increase in the number of excitatory synapses, along with a corresponding decrease in the percentage of presynaptically silent synapses during neuronal development. To pinpoint the distribution of presynaptically active and silent synapses, i.e., their positional information, we employed Sholl analysis. Our results indicate that the distribution of presynaptically silent synapses within a single neuron does not exhibit a distinct pattern during synapse development in different distance from the cell body. However, irrespective of neuronal development, the proportion of presynaptically silent synapses tends to rise as the projection site moves farther from the cell body, suggesting that synapses near the cell body may exhibit higher synaptic transmission efficiency. This study represents the first observation of changes in the distribution of presynaptically active and silent synapses within a single neuron.

## 1 Introduction

An excitatory synapse releases glutamate as a neurotransmitter, and it is referred to as an active synapse. Conversely, a synapse that maintains its synaptic structure but fails to transmit neuronal information is known as a silent synapse (Durand et al., 1996). Silent synapses can become inactive for one of two reasons: (I) the absence or impairment of receptor function in the post-synaptic membrane or (II) the loss of synaptic exocytotic function in the nerve terminal.

Regarding reason (I), the relationship between two types of glutamate receptors, namely the N-methyl-D-aspartate (NMDA) receptor and the α-amino-3-hydroxy-5-methyl-4-isoxazolepropionic acid (AMPA) receptor, is speculated as follows: in the immature brain shortly after birth, NMDA receptors are expressed but blocked by Mg^2+^. Even when neurotransmitters are released and glutamate is received, NMDA receptors remain inactive, and neuronal information is not transmitted to subsequent neurons (Kerchner and Nicoll, 2008; Hanse et al., 2013). However, as the brain matures, AMPA receptors appear near NMDA receptors. When released glutamate binds to AMPA receptors, depolarization occurs, removing the magnesium block at the NMDA receptor, leading to the activation of the synapse (Itami et al., 2003). Consequently, the nerve cell becomes more excited and transmits information to the next cell. In contrast to the mechanism described above, the physiological significance of reason (II) has not been fully elucidated (Moulder et al., 2008).

*In vitro* conditions allowed for electrophysiological and morphological analyses, revealing notable distinctions in neuronal synaptogenesis (Fletcher et al., 1991; Basarsky et al., 1994; Gottmann et al., 1994; Mohrmann et al., 2003). Building upon widely accepted concepts concerning presynaptic synaptogenesis, this study delves into presynaptic synaptogenesis within cultures spanning 1 week, 2 weeks, and 3 weeks. Our investigation also places particular emphasis on the distance from the cell body in relation to the proportion of presynaptically active and silent synapses projecting to dendrites.

## 2 Materials and methods

### 2.1 Animal ethics

All animal care procedures followed the rules of the Fukuoka University Experimental Animal Welfare Committee (equivalent to NIH guidelines). The experiment was strictly conducted after the Committee’s approval of the experimental plan. Cultured cells were obtained by decapitating newborn mice, and efforts were made to minimize distress.

All experiments were performed in compliance with the ARRIVE guidelines. Experiments were performed blind.

### 2.2 Experimental animals

Timed-pregnant Jcl:ICR mice (Catalog ID: Jcl:ICR, CLEA Japan, Inc., Tokyo, Japan) were purchased at gestational day 15 from the Kyudo Company (Tosu, Japan). Fifteen to seventeen-week-old pregnant Jcl:ICR mice were used. The pregnant mice were housed in plastic cages in an environment with a room temperature of 23 ± 2°C, a humidity of 60 ± 2%, and a 12 h light-dark cycle (lights on at 7:00 AM, lights off at 7:00 PM). Food (CLEA Rodent Diet, CE-2, CLEA Japan, Inc., Tokyo, Japan) and water were provided *ad libitum*. The body weights of pregnant mice were not recorded.

Experimental animals were handled in accordance with the animal ethics regulations of the Fukuoka University Animal Care and Use Committee (Approval Nos. 2112094 and 2311081).

### 2.3 Autaptic culture preparation

A sample in which a single neuron is cultured on a dot-like layer of astrocytes is referred to as an autaptic culture (Bekkers and Stevens, 1991). The autaptic culture preparations were conducted in accordance with previous reports (Bekkers and Stevens, 1991; Kawano et al., 2012; Oyabu et al., 2020). To provide a brief overview, postnatal day 0–1 neonatal mice were used, and their brains were extracted and immersed in Hank’s Balanced Saline Solution (Thermo Fisher Scientific, Waltham, MA, United States, Cat. # 084-08345) cooled to 4°C. In this state, the cerebral cortices on both sides were excised under a microscope, and cerebral cortical cells were isolated via trypsinization. The isolated cells were then cultured with Dulbecco’s modified Eagle’s medium (DMEM) with GlutaMAX-I and pyruvate (Thermo Fisher Scientific, Waltham, MA, United States), supplemented with 10% fetal bovine serum (Thermo Fisher Scientific, Waltham, MA, United States) and 0.1% MITO + Serum Extender (BD Biosciences, San Jose, CA, USA) in 75 cm^2^ culture flasks (Corning Inc., NY, United States). After 2 weeks, the culture flask was gently tapped multiple times to remove non-astrocytic cells. Subsequently, the astrocytes that remained in close contact with the bottom of the culture flask were detached using trypsinization. These cells were replated at a density of 6,000 cells/cm^2^ per well onto 22 mm round coverslips (thickness No. 1; Matsunami, Osaka, Japan) within 6-well plates (TPP, Trasadingen, Switzerland).

To cultivate the seeded astrocytes in dot shapes, a mixture of collagen (final concentration 1.0 mg/mL; BD Biosciences, San Jose, CA, United States) and poly-D-lysine (final concentration 0.25 mg/mL; Sigma-Aldrich, St Louis, MO, United States) was prepared. Subsequently, 300 μm square dots were stamped onto a round cover glass pre-coated with 0.5% agarose. This stamp design was an original development (Kawano et al., 2012; Oyabu et al., 2020). One week after seeding the astrocytes, it was confirmed that the astrocytes had successfully formed dot-shaped cultures.

Next, brains were excised from neonatal ICR mice on days 0–1 after birth and immersed in Hank’s Balanced Saline Solution cooled to 4°C. In this state, the hippocampal CA3–CA1 region was dissected under a microscope. Finally, hippocampal neurons were isolated through treatment in Dulbecco’s modified Eagle’s medium (Invitrogen) containing 2 U/mL of papain (Worthington, Cat. # PAP) at 37°C for 1 h. The isolated hippocampal neurons were then seeded at a density of 1,500 cells/cm^2^ per well and cultured with serum-free Neurobasal-A medium (Thermo Fisher Scientific, Waltham, MA, United States), supplemented with 2% B27 supplement (Thermo Fisher Scientific, Waltham, MA, United States) in a 37°C, 5% CO_2_ incubator. Data from three groups (1 week, 2 weeks, and 3 weeks *in vitro*, respectively) were obtained from the same sister cultures (15 cultures in total).

### 2.4 FM1-43FX dye staining

Presynaptic terminals that actively release neurotransmitters, referred to as active synapses, were visualized using N-(3-triethylammoniumpropyl)-4-(4-(dibutyl amino) styryl) pyridinium dibromide (FM1-43FX, a fixable analog of FM1-43 membrane stain, Thermo Fisher Scientific, Waltham, MA, United States). To stain the presynaptically active synapses of autaptic cultured neurons, we followed the method of Moulder et al. (2008, 2010). In brief, we dissolved 10 μM FM1-43FX in a high potassium (45 mM) extracellular solution containing the NMDA receptor inhibitor (2R)-amino-5-phosphonovaleric acid (APV, 25 μM, Sigma-Aldrich, St Louis, MO, United States) and the AMPA receptor inhibitor 6-cyano-7-nitroquinoxaline-2,3-dione (CNQX, 10 μM, Sigma Aldrich, St Louis, MO, United States). This solution was applied to the autaptic culture neurons for 2 min. Subsequently, the cells were washed three times for 2 min each with a standard extracellular solution containing 1 μM tetrodotoxin (TTX), a sodium channel blocker.

Following the staining procedure, autaptic culture neurons were fixed using a 4% paraformaldehyde solution in phosphate-buffered saline (PBS) for 10 min. To minimize the loss of FM1-43FX signals, such as photobleaching due to ambient light exposure, the images were captured promptly after fixing the neurons. We acquired 16-bit images using an all-in-one fluorescence microscope (BZ-X810, KEYENCE, Osaka, Japan) with a 20× objective lens (Plan Apochromat, numerical aperture 0.75), or an sCMOS camera (pco.edge 4.2, pco, Kelheim, Germany) mounted on an inverted microscope (Eclipse-TiE, Nikon, Tokyo, Japan) equipped with a 40× objective lens (Plan Apoλ, numerical aperture 0.95). In the case of using the inverted microscope, FM1-43FX was excited using a white LED (Lambda HPX, Sutter Instruments, Novato, CA, United States) at 100% maximum intensity and imaged using a filter cube (470/40 nm excitation, 500 nm dichroic long-pass, 535/50 nm emission). In each sample, 10 images were captured with an exposure time of 300 ms per image, averaged, and utilized for analysis based on the average pixel intensity.

### 2.5 Immunostaining

Autaptic culture preparations underwent immunostaining based on the method established by Moulder et al. (2008, 2010). After capturing FM1-43FX images, autaptic culture neurons were incubated in a microscope chamber with PBS containing 5% normal goat serum and 0.1% Triton X-100 (Sigma Aldrich, St Louis, MO, United States) for 30 min. Following the Triton X-100 blocking step, the decolorization of FM1-43FX was visually confirmed (data not shown). Primary antibodies were subsequently applied for 3 h at the following dilutions: anti-microtubule-associated protein 2 (MAP 2) at 1:1,000 (guinea pig polyclonal, antiserum, Synaptic Systems, Göttingen, Germany) and anti-vesicular glutamate transporter 1 (anti-vGLUT1) at 1:2,000 (rabbit polyclonal, affinity-purified, Synaptic Systems, Göttingen, Germany). Secondary antibodies were applied using Alexa Fluor 488 or 594 (Thermo Fisher Scientific, Waltham, Mass., United States) at a dilution of 1:400 for 30 min. Since FM1-43FX can be completely removed by Triton X-100 blocking (Moulder et al., 2008; Oyabu et al., 2020), the excitation light (480 nm) used for fluorescence observation of FM1-43FX was also employed for fluorescence excitation of Alexa Fluor 488.

Imaging of autaptic culture preparations was performed using an all-in-one fluorescence microscope (BZ-X810, KEYENCE, Osaka, Japan) with a 20× objective lens (Plan Apochromat, numerical aperture 0.75), or an sCMOS camera (pco.edge 4.2, pco, Kelheim, Germany) mounted on an inverted microscope (Eclipse-TiE, Nikon, Tokyo, Japan) equipped with a 40× objective lens (Plan Apoλ, numerical aperture 0.95). Similar to FM1-43FX imaging, 10 images were captured per sample, and these images were subsequently normalized to obtain the average intensity for analysis.

### 2.6 Qualification of synaptic puncta

To identify the vGLUT1 puncta, we employed ImageJ software (version 1.46j; Rasband, W.S., ImageJ, U. S. National Institutes of Health, Bethesda, Maryland, United States, https://imagej.nih.gov/ij/, 1997–2016). We subtracted the original images from an image filtered with a Gaussian blur of the duplicated original image. For detailed procedures, please refer to Iwabuchi et al. (2014). The subtracted images were then subjected to binarization using a threshold set at the top of 0.01% of the cumulative intensity of the background area in the vGLUT1 image. Subsequently, we detected the number of puncta overlaid with MAP2 images, applying a size threshold of ≥5 pixels.

### 2.7 Qualification of presynaptically silent synapses

**Figure 1:**
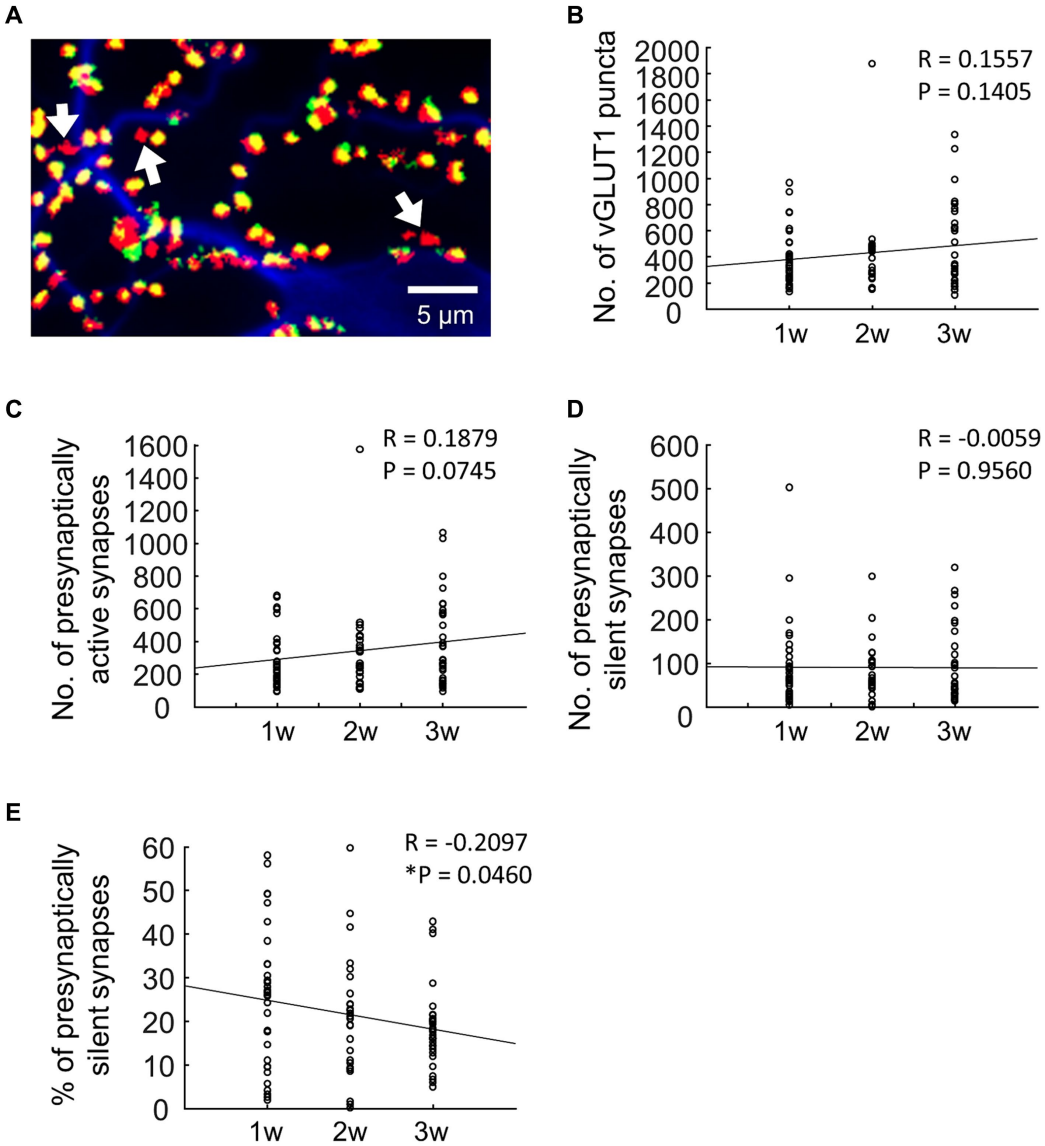
Changes in the number of synapses during development. **(A)** Typical fluorescence image. In terms of pseudocolors, vGLUT1 is labeled in red, FM1-43FX is in green, and MAP2 is in blue. Therefore, when the green staining of FM1-43FX and the red staining of vGLUT1 overlap, presynaptically active synapses appear yellow. Conversely, presynaptically silent synapses lack the green label and are denoted as red-only puncta by vGLUT1 (as indicated by the arrows in **A**). **(B)** Quantification of the number of vGLUT1-positive synapses (blue bar: 1 w: *n* = 33, orange bar: 2 w: *n* = 26, gray bar: 3 w: *n* = 32). **(C)** Quantification of the number of presynaptically active synapses (blue bar: 1 w: *n* = 33, orange bar: 2 w: *n* = 26, gray bar: 3 w: *n* = 32). Data were obtained from the same neuron as in **B**. **(D)** Quantitation of the number of presynaptically silent synapses (blue bar: 1 w: *n* = 33, orange bar: 2 w: *n* = 26, gray bar: 3 w: *n* = 32). Data were obtained from the same neuron as in **B**. **(E)** Percentage of presynaptically silent synapse numbers (blue bar: 1 w: *n* = 33, orange bar: 2 w: *n* = 26, gray bar: 3 w: *n* = 32). Data were obtained from the same neuron as in **B**.

The FM1-43FX puncta were identified in a manner similar to that employed for vGLUT1 puncta. We overlaid images of FM1-43FX with images of vGLUT1 and MAP2 to identify presynaptically silent synapses (Figure 1A). Utilizing ImageJ, we defined the region of interest (ROI) of vGLUT1 that was not stained with FM1-43FX as a presynaptically silent synapse. For a more comprehensive understanding of the analysis of silent synapses, please refer to our previous study (Oyabu et al., 2020).

### 2.8 Sholl analysis

**Figure 2:**
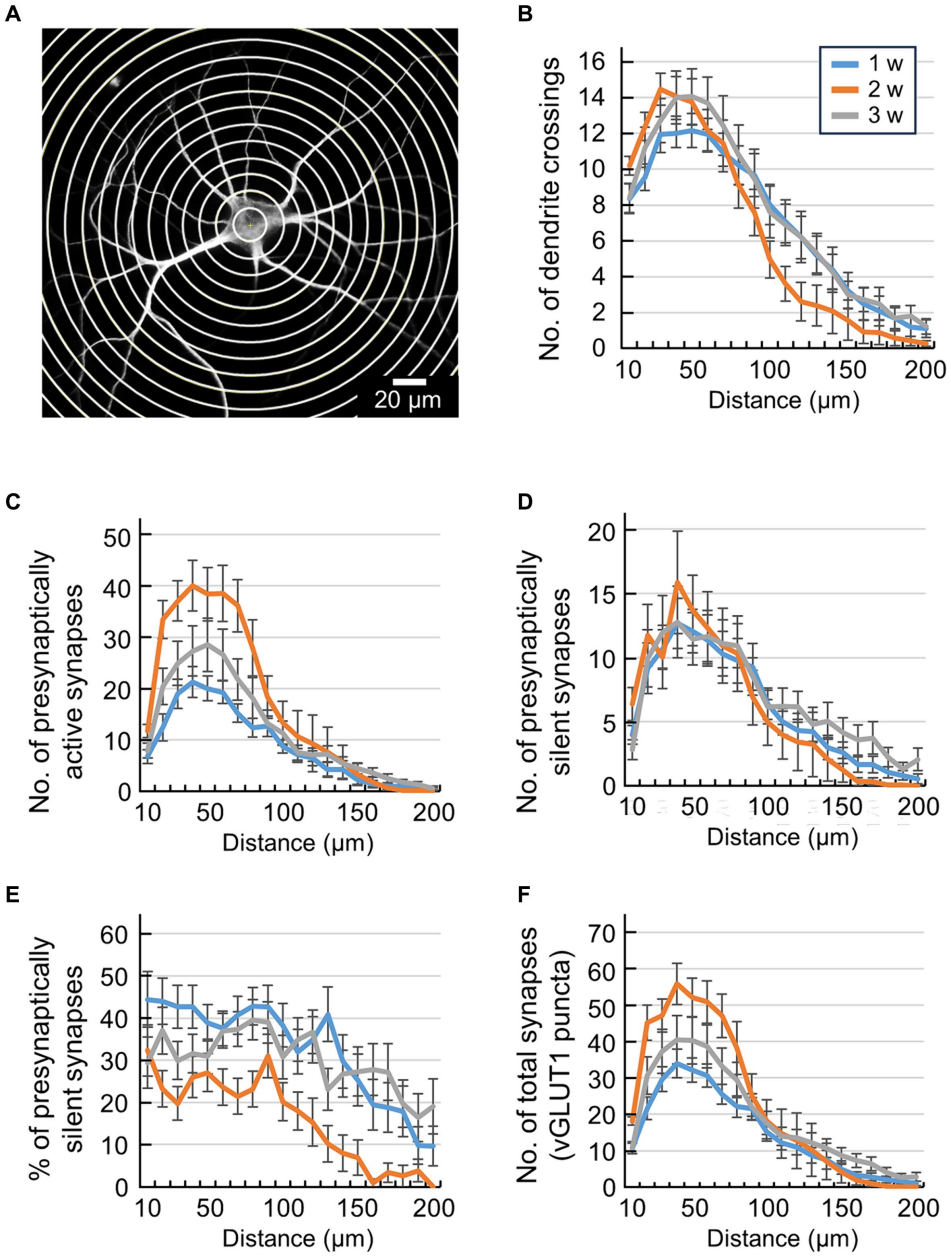
Quantification of synaptic location information by conventional Sholl analysis. **(A)** Scheme of Sholl analysis of a single hippocampal neuron. Concentric circles were drawn at 10 μm intervals around the cell body. **(B)** Quantification of the number of dendrite crossings in concentric circles (blue line: 1 w: *n* = 33, orange line: 2 w: *n* = 26, gray line: 3 w: *n* = 32). The *x*-axis indicates the distance from the center of cell body up to 200 μm. Data were obtained from the same neuron as in Figure 1B. Significant effect of Week [*F*(2,1720) = 6.4, *p* < 0.001] and Distance [*F*(19,1720) = 55.03, *p* < 0.001] but not interaction [*F*(38,1720) = 0.96, *p* > 0.53], two-way ANOVA. **(C)** Quantification of the number of presynaptically active synapses in each area (blue line: 1 w: *n* = 33, orange line: 2 w: *n* = 26, gray line: 3 w: *n* = 32). The *x*-axis indicates the distance from the center of the cell body up to 200 μm. Data were obtained from the same neuron as in Figure 1B. Significant effect of Week [*F*(2,1819) = 38.17, *p* < 0.001], Distance [*F*(19,1819) = 43.41, *p* < 0.001] and interaction [*F*(38,1819) = 2.3, *p* > 0.001], two-way ANOVA. **(D)** Quantification of the number of presynaptically silent synapses in each area (blue line: 1 w: *n* = 33, orange line: 2 w: *n* = 26, gray line: 3 w: *n* = 32). The *x*-axis indicates the distance from the center of the cell body up to 200 μm. Data were obtained from the same neuron as in Figure 1B. No significant effect of Week [*F*(2,1819) = 2.9, *p* > 0.05], significant effect of Distance [*F*(19,1819) = 20.72, *p* < 0.001] and no interaction [*F*(38,1819) = 0.43, *p* > 0.99], two-way ANOVA. **(E)** Percentage of presynaptically silent synapse numbers in each area (blue line: 1 w: *n* = 33, orange line: 2 w: *n* = 26, gray line: 3 w: *n* = 32). The *x*-axis indicates the distance from the center of the cell body up to 200 μm. Data were obtained from the same neuron as in Figure 1B. Significant effect of Week [*F*(2,1819) = 57.93, *p* < 0.001] and Distance [*F*(19,1819) = 9.63, *p* < 0.001] but not interaction [*F*(38,1819) = 0.89, *p* > 0.66], two-way ANOVA. **(F)** Quantification of the number of total synapses (vGLUT1-positive puncta) in each area (blue line: 1 w: *n* = 33, orange line: 2 w: *n* = 26, gray line: 3 w: *n* = 32). The *x*-axis indicates the distance from the center of the cell body up to 200 μm. Data were obtained from the same neuron as in Figure 1B. Significant effect of Week [*F*(2,1819) = 21.05, *p* < 0.001], Distance [*F*(19,1819) = 49.24, *p* < 0.001] and interaction [*F*(38,1819) = 1.79, *p* < 0.001], two-way ANOVA.

The Sholl analysis plugin (Sholl, 1953) within Image J was employed to examine the projection positions of presynaptically silent synapses. The methodology for this analysis is outlined as follows: initially, a minimum circle with a diameter of 10 μm was delineated around the cell body. Subsequently, concentric circles with increasing radius (increments of 10 μm) were placed around the soma until the entire MAP2 image was encompassed (Figure 2A). Then, the number of crossing dendrites was counted in each circle of the Sholl analysis. The number of synaptic puncta along dendrites was counted between two subsequent circles of the Sholl analysis (Schmitz et al., 2011; Rotterman et al., 2014; Galati et al., 2016).

**Figure 3:**
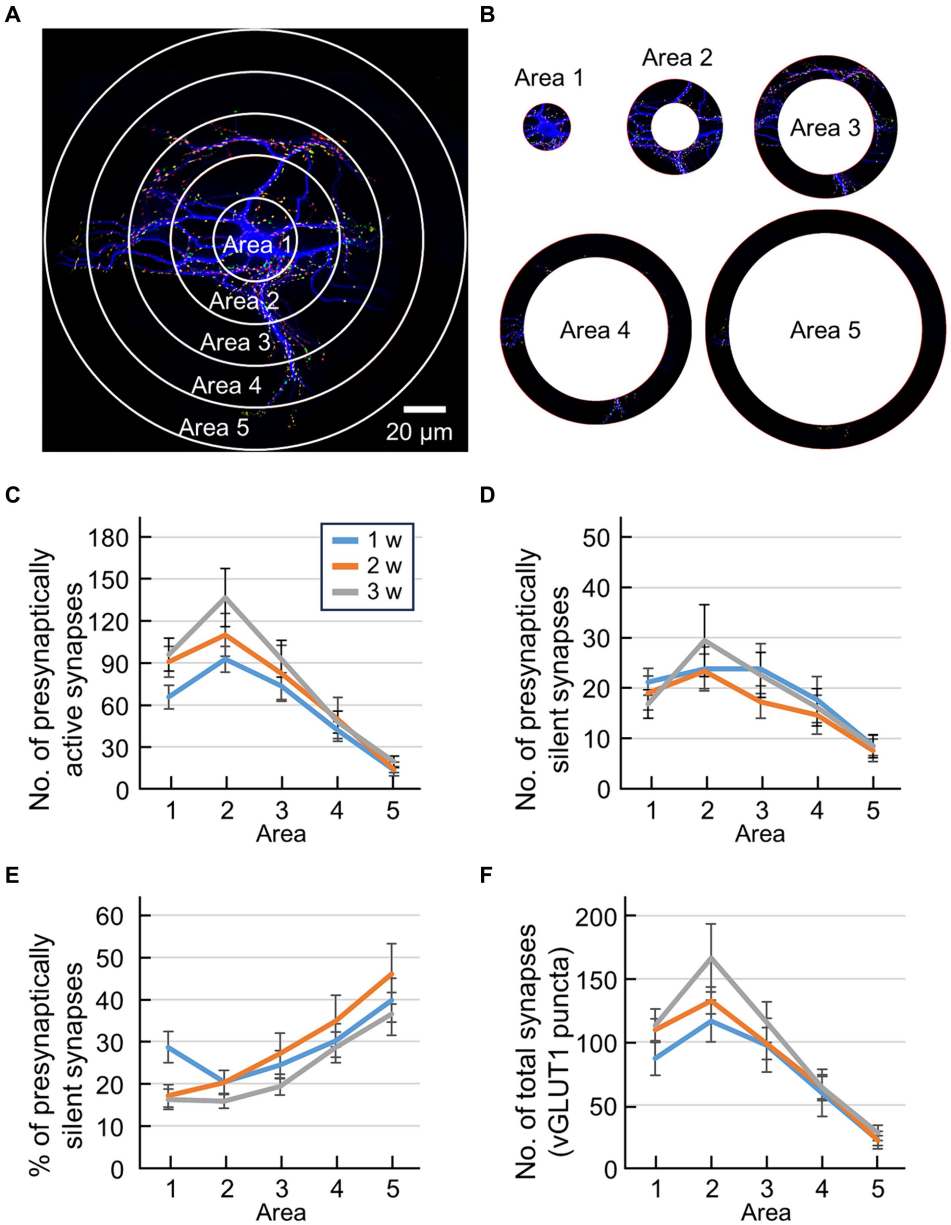
Quantification of synaptic location information by modified Sholl analysis. **(A)** Fluorescence image of an autaptic culture preparation before sectioning into 5 areas using Sholl analysis. The inside of the minimal circle is area 1, the area from the outside of the minimum circle to the next concentric circle is area 2, the area to the next concentric circle is area 3, the area to the next concentric circle is area 4, and the outermost area is area 5. **(B)** Sectioned area cut away. **(C)** Quantification of the number of presynaptically active synapses in each area (blue line: 1 w: *n* = 33, orange line: 2 w: *n* = 26, gray line: 3 w: *n* = 32). The horizontal axis indicates the area number. Data were obtained from the same neuron as in Figure 1B. Significant main effect of Area [*F*(4,438) = 31.14, *p* < 0.001] and Weeks [*F*(2,438) = 4.39, *p* < 0.02], and no significant interaction [*F*(8,438) = 0.62, *p* > 0.75], two-way ANOVA. Significant differences between area 2 and area 5, for 1, 2 and 3 weeks, and area 2 and area 4 for 2 and 3 weeks (post-hoc HSD test, *p* < 0.05). **(D)** Quantification of the number of presynaptically silent synapses in each area (blue line: 1 w: *n* = 33, orange line: 2 w: *n* = 26, gray line: 3 w: *n* = 32). The horizontal axis indicates the area number. Data were obtained from the same neuron as in Figure 1B. Significant main effect of Area [*F*(4,438) = 7.7, *p* < 0.001], and no significant interaction [*F*(8,438) = 0.33, *p* > 0.95], two-way ANOVA. No significant difference by post-hoc HSD test. **(E)** Percentage of presynaptically silent synapse numbers per area (blue line: 1 w: *n* = 33, orange line: 2 w: *n* = 26, gray line: 3 w: *n* = 32). The horizontal axis indicates the area number. Data were obtained from the same neuron as in Figure 1B. Significant main effect of Area [*F*(4,438) = 15.11, *p* < 0.001] and Weeks [*F*(2,438) = 3.32, *p* < 0.04], and no significant interaction [*F*(8,438) = 0.8, *p* > 0.60], two-way ANOVA. Significant differences between area 1 and area 5, for 2 and 3 weeks, and area 2 and area 5 for 2 and 3 weeks (post-hoc HSD test, *p* < 0.05). **(F)** Quantification of the number of total synapses (vGLUT1-positive puncta) in each area (blue line: 1 w: *n* = 33, orange line: 2 w: *n* = 26, gray line: 3 w: *n* = 32). The horizontal axis indicates the area number. Data were obtained from the same neuron as in Figure 1B.

It is difficult to compare the developmental features of synaptic distribution using conventional Sholl analysis because dendrites spread with individual capability and neuronal development. Therefore, we compared the developmental changes in synaptic distribution relatively by keeping the number of concentric circles constant (Figure 3). In this analysis, an inner circle with a diameter of 20 μm was delineated around the cell body. Concentric circles were drawn to encompass the entire MAP2 image, including three additional concentric circles positioned between the maximum circle and the central minimum circle (Figure 3A). Note that the diameter of the innermost circle was always 20 μm, while the spacing between the concentric circles varied among neurons. The region inside the minimal circle was designated as area 1, the region between the outer edge of the minimum circle and the subsequent concentric circle was labeled as area 2, the area extending to the next concentric circle was designated as area 3, the region encompassing the following concentric circle was defined as area 4, and the outermost region was identified as area 5 (Figure 3B). By categorizing these 5 areas, it became possible to compare the synapse positions between different sized neurons during neuronal development.

Only neurons located in the center of the astrocytic island were used for data analysis to avoid dendrites and axons projecting in a biased direction and altering their normal extension.

### 2.9 Statistics

Data are expressed as mean ± SEM. Statistical tests were conducted using Matlab Statistics Toolbox (MathWorks, Natick, MA). Developmental changes of total number of synapses or total proportion of synapses were evaluated using Pearson correlation coefficients between the culture period and number of synapses or the culture period and proportion of synapses, respectively (Figures 1B–E). We evaluated changes in synapse numbers and proportions based on distance from the soma using a two-way analysis of variance with Distance and Week as factors (Figures 2B–F). Developmental changes in numbers and proportions of synapses across areas were evaluated using a two-way analysis of variance, with Area and Week as factors, followed by Tukey’s HSD test (Figures 3C–E). The threshold for statistical significance was set at *p* < 0.05.

## 3 Results

### 3.1 Quantification of presynaptically active and presynaptically silent synapses

FM1-43FX is internalized into the presynaptic terminals through the process of synaptic vesicle endocytosis. Consequently, we used fluorescent puncta labeling to identify presynaptically active synapses capable of neurotransmitter exocytosis. Figure 1A illustrates a representative fluorescence image in which vGLUT1 is pseudocolored in red, FM1-43FX in green, and MAP2 in blue. In the case of presynaptically active synapses, the overlap of the green FM1-43FX and red vGLUT1 stains results in a yellow appearance, indicating the presence of a presynaptically active synapse. Conversely, presynaptically silent synapses, which fail to uptake FM1-43FX, are not marked in green but appear solely as red puncta by vGLUT1 (indicated by arrows in Figure 1A). Essentially, the red fluorescent puncta denote presynaptically silent synapses, which are excitatory synapses that do not release glutamate through exocytosis.

First, we quantified the number of synapses positive for the vGLUT1 antibody. The number of excitatory synapses increased gradually as neurons developed (Figure 1B, 1 w: 381.58 ± 36.63, 2 w: 426.58 ± 62.55, 3 w: 486.56 ± 57.11; *R* = 0.16, *p* = 0.14). It is important to note that the vGLUT1 puncta in this result represents both presynaptically active and presynaptically silent synapses. In other words, the presence of the glutamatergic marker vGLUT1 in any part of the field shows that even presynaptically silent synapses can still be identified as glutamatergic synapses.

To specifically quantify presynaptically active synapses, we counted the number of synapses labeled with FM1-43FX among vGLUT1-positive synapses. As neurons developed, there was a gradual increase in the number of presynaptically active synapses (Figure 1C, 1 w: 287.09 ± 29.42, 2 w:347.65 ± 54.92, 3 w: 393.13 ± 46.25); *R* = 0.19, *p* = 0.075.

Next, we quantified presynaptically silent synapses among vGLUT1-positive synapses, identified as those where FM1-43FX was not labeled (Figure 1D). The results revealed no change in the number of presynaptically silent synapses with neuronal development (Figure 1D, 1 w: 94.48 ± 16.76, 2 w: 81.88 ± 12.87, 3 w: 93.44 ± 14.93; *R* = −0.0059, *p* = 0.96). Based on these findings, we calculated the ratio of presynaptically silent synapses (Figure 1E) and observed that the proportion significantly decreased with neuronal development (Figure 1E, 1 w: 25.04 ± 2.75%, 2 w: 20.91 ± 2.73%, 3 w: 18.45 ± 1.61%; *R* = −0.2097, *p* < 0.05).

### 3.2 Location analysis of synapses using Sholl analysis

Upon observing the image in Figure 1A, we noted that the projection positions of presynaptically active and silent synapses onto the dendrites exhibited uneven distribution. Consequently, we endeavored to quantify the positional information of these synapses within a single neuron. To conduct this positional analysis of synaptic puncta, we employed the conventionally known Sholl analysis (Sholl, 1953). Sholl analysis is a widely used and straightforward method for quantifying the branching patterns of dendrites and axons. The MAP2 image in Figure 2A shows an autaptic culture with concentric circles drawn in 10 μm steps. The Sholl analysis counted the number of intersections between concentric circles and dendrites up to 200 μm from the center of the cell body (Figure 2B). Two-way ANOVA revealed the significant effect of Distance [*F*(2,1720) = 6.4, *p* < 0.001] and Week [*F*(19,1720) = 55.0, *p* < 0.001] but not the interaction [*F*(38,1720) = 0.96, *p* > 0.53]. This finding suggests that the number of dendritic branching decreased along the distance, but the pattern of dendritic branching did not alter with development. This is likely due to the narrow and limited area of the astrocyte island making it difficult to discern differences between the culture periods. Next, using Sholl analysis, we counted the number of glutamatergic synapses between concentric circles in 10 μm steps (Figures 2C–F). Two-way ANOVA revealed the significant effect of Week in all the synapse counts (*p* < 0.05; see Figure 2 legend for details of statistics) except the number of presynaptically silent synapses (*p* > 0.055, Figure 2D). This statistical result shows that the number of presynaptically active synapses increased during development (Figure 2C). The majority of glutamatergic synapses were formed during the second week of culture. Two-way ANOVA also revealed the significant effect of Distance in all the synapse counts (*p* < 0.05; see Figure 2 legend for statistics details). This result suggests that the synapse count decreased along the distance. A higher density of synapses in dendrites was observed at a distance of around 50 μm from the center of the cell body. Significant interactions between Distance and Week were found for number of presynaptically active synapses (*p* < 0.001, Figure 2C) and number of total vGLUT1 puncta (*p* < 0.001, Figure 2F). This result indicates that the distributions of the synapse count along dendrites changed during development. The data shows that within a radius of up to 100 μm from the cell body, both the number of presynaptically active synapses and the number of vGLUT1 puncta decreased in the third week of culture (Figures 2C,F). No significant interactions between Distance and Week were found for the number of presynaptically silent synapses (*p* > 0.99, Figure 2D) or the percentage of presynaptically silent synapses (*p* > 0.66. Figure 2E).

Individual differences of dendritic extent are large because single neurons develop in a limited area of an astrocyte island. For example, neurons that can form dendrites far from the cell body can also form synapses far from the cell body. In contrast, neurons that complete dendrite formation close to the cell body cannot form synapses far from the cell body. A neuron’s synaptic distribution may be biased if the absolute distances from the cell body are different and are averaged as one group. That is, in Sholl analysis based on the “real distance” from the cell body, the number of data decreases for concentric circles farther from the cell body, and if we count as zero the data from neurons whose dendrite distribution has ended, the averaged value statistically approaches zero (Figures 2C–F). Thus, conventional Sholl analysis cannot objectively evaluate how the percentage of presynaptically active/silent synapses changes with distance from the cell body. We compared the developmental changes in synaptic distribution by keeping the number of concentric circles constant, indexed by “relative distance” from the cell body, to avoid such defects. In this analysis, concentric circles were drawn around the neuron’s cell body (Figure 3A), and these concentric circles were then divided into 5 areas (Figure 3B). Synapses were tallied within each of these regions, enabling precise quantification of the synapse distribution (Figures 3C–F).

The number of presynaptically active synapses in each area exhibited a peak in area 2 for all three groups (Figure 3C). Two-way ANOVA using Area and Weeks as factors revealed significant effects for both Area [*F*(4,438) = 31.14, *p* < 0.001] and Weeks [*F*(2,438) = 4.39, *p* < 0.02]. This indicates that the number of presynaptically active synapses decreased along dendritic distance and increased along the course of development. Post-hoc HSD test further revealed significant differences between area 2 and area 5 for all three groups, and between areas 2 and 4 for 2 and 3 weeks (*p* < 0.05). This indicates that the number of presynaptically active synapses decreased as the distance from the cell body increased (Figure 3C). Similar patterns are observed in the number of total synapses (Figure 3F). For the number of presynaptically silent synapses, two-way ANOVA using Area and Weeks as factors revealed a significant main effect of Area [*F*(4,438) = 7.7, *p* < 0.001], however, no significant difference was found in the post-hoc HSD test (Figure 3D). To analyze the distribution of presynaptically silent synapses further, we calculated the percentage of presynaptically silent synapses for each area (Figure 3E). Two-way ANOVA using Area and Weeks as factors revealed significant main effects of both Area [*F*(4,438) = 15.11, *p* < 0.001] and Weeks [*F*(2,438) = 3.32, *p* < 0.04]. This suggests that the percentage of presynaptically silent synapses increased along the dendritic distance. Intriguingly, post-hoc HSD test further revealed that the proportions of presynaptically silent synapses near the cell body (area 1 and area 2) and that of the most distal part of the cell body (area 5) were significantly different for 2 and 3 weeks (*p* < 0.05) but not for 1 week, indicating developmental change of the distribution of presynaptically silent synapses (Figure 3E).

## 4 Discussion

Autaptic cultures are a simplified form of synaptic organization, consisting of a single closed-loop neuronal circuit. Although they may have some disadvantages compared to *in vivo* neuronal networks, they have advantages that can help answer fundamental questions about synaptic transmission, organization, and development.

Autaptic cultures are thought to have less preserved synaptic features than normal dissociated cultures or more intact preparations. This raises questions about the applicability of findings made in the autaptic culture experiments as common neuronal properties. However, many experimental findings made with autapses have been well translated to the more physiological experimental conditions or even to the intact brain. It has been reported that autapses in single neuron cultures and synapses in dissociated neuron cultures share similar biophysical properties (Bekkers and Stevens, 1991; Shi and Rayport, 1994). In addition, previous studies of the synaptic vesicle cycle at autapses (Chamberland and Tóth, 2016) and synaptic plasticity (Goda and Stevens, 1996; Straiker and Mackie, 2005; Kellogg et al., 2009; Rost et al., 2010) indicate the similarity between autapses and synapses in the intact brain.

The autaptic culture’s two-dimensional structure has technical advantages because it allows easy access to individual cells for electrical recording and high-visibility optical imaging. Autaptic neurons exhibit morphological development of dendrites and spontaneous firing similar to neurons within intact brain circuits. In culture, an autaptic neuron is innervated by a single afferent originating from itself. Thus, the isolated environment of an autaptic neuron has advantages in correlating the pure relationship between neuronal activity and synaptic development along the dendritic trees.

Compared to a previous study (Rosenmund et al., 2002), where the percentage of active synapses was approximately 66%, our experiments revealed percentages ranging from about 75–80% (Figure 1E). Clearly, these disparities can be attributed to extrinsic factors such as the culture conditions of the neurons, though differences in experimental methods cannot be discounted. We specifically evaluated presynaptically active/silent synapses using FM1-43X endocytosis. However, given that synapses can be considered active upon vesicle release, the definition of “active” might consider whether the FM1-43FX fluorescence, captured during the initial stimulation, decreases during the second stimulation. It is important to note that a similar concern applies to the original paper discussing presynaptically silent synapses (Moulder et al., 2010).

In the previous study (Rosenmund et al., 2002), FM dye staining was conducted using action potential trains and treated with a high potassium solution for FM dye-destaining. Our present experiment employed robust stimuli, a high-potassium solution for FM1-43FX staining. It is plausible that such a potent stimulus may have “awakened” dormant presynaptic synapses. Therefore, measuring functional active presynapses through electrical stimulation is an avenue for future investigation. In addition, while neuronal activity patterns do influence synaptogenesis, notably, autaptic neurons do not form intricate networks with other neurons. Furthermore, the activity patterns in autaptic neurons are undoubtedly distinct from those observed in conventional dense networks. For example, contact inhibition is limited in autapse cultures. Contact inhibition is a phenomenon observed in cells, particularly in cell culture and tissue growth, where cells stop dividing or migrating upon coming into contact with neighboring cells. Essentially, it’s a mechanism that prevents cells from overpopulating or spreading uncontrollably. Contact inhibition enables the regulation of the density and distribution of synaptic connections between neurons, particularly during synapse formation. Neurons often form synaptic contacts with specific target cells, and contact inhibition may help ensure that these connections are appropriately spaced and distributed to facilitate efficient neuronal communication. Consequently, the impact of activity patterns on presynaptically silent synapses should also be examined in future studies.

The increase in the number of excitatory synapses with neuronal development is a well-documented phenomenon (Brewer et al., 2009; Ito et al., 2013), and the findings of our study align with this observation (Figure 1B). Turning attention to the percentage of presynaptically silent synapses within each compartment, we observed that approximately 20% remained silent in areas 2–3, while approximately 40% were silent in area 5, indicating that silent synapses tend to form at a greater distance from the cell body. This suggests a trend toward an increased presence of excitatory active synapses proximal to the soma (Figure 3C). Synapses in close proximity to the cell body were posited to be more active than those distal to the cell body during neuronal development, with potential explanations. For example, as synapses develop, they undergo synaptic pruning, a process where axons reshape neuron dendrites and synapses, eliminating unnecessary synapses during brain development (Lichtman and Colman, 2000; Hua and Smith, 2004; Kano and Hashimoto, 2009; Riccomagno and Kolodkin, 2015). The process of synaptic pruning has been studied extensively, and it has been found that both neural activity that is dependent on development and changes in neural activity due to sensory experiences are crucial for the creation of central nervous system circuits (Faust et al., 2021). For instance, the critical period when synaptic pruning happens in the cerebral cortex refers to a limited postnatal period of increased plasticity in neural networks. During this critical period, two different types of synaptic models have been proposed: “innate synapses,” which build rudimentary networks with innate functions, and “gestalt synapses,” which govern experience-dependent refinement features (Xu et al., 2020). The nascent gestalt synapse is always formed as an AMPA receptor-silenced synapse, which serves as a substrate for critical period plasticity. Although there have been reports on the relationship between postsynaptically silent synapses and synaptic pruning, the causal relationship between these two remains unknown. Further research is needed to pursue the physiological significance of presynaptic silent synapses.

In this study, we did not observe significant changes in the number of presynaptically silent synapses during development (Figure 1D). It is worth noting that changes in the number of postsynaptically silent synapses during development have been reported (Rumpel et al., 2004). For instance, in the neonatal rat visual cortex, many silent synapses exist in layer VI pyramidal neurons, and the number of active synapses increases with growth, similar to the hippocampus. On the other hand, layer II/III pyramidal cells have many active synapses at birth, and silent synapses increase with growth, followed by a return to active synapses (Rumpel et al., 2004). Thus, the patterns of developmental post-synaptic expression appear to vary by brain region. It remains unclear whether the results of this study are specific to glutamatergic neurons in the hippocampus or if similar patterns are observed in other brain regions.

While no distinct changes were observed in the ratio of silent synapses in each area during neuronal development, an interesting finding was that, regardless of neuronal maturation, the proportion of presynaptically silent synapses was lower in the proximal region compared to the distal region of the cell body. Although the physiological significance of changes in the rate of presynaptically silent synapse formation during neuronal development remains unknown, it may contribute to the establishment of functional neural circuits.

Presynaptically silent synapses, despite being structurally mature, are believed to lack neurotransmitter release due to the inability of synaptic vesicles to exocytose (Faust et al., 2021). Several proteins, such as Munc13, RIM, CAST, and bassoon, are involved in neurotransmitter exocytosis from nerve terminals (Südhof, 2012; Mochida, 2022). Among these, Rim1 and Munc13-1 have been reported to decrease in expression after the induction of silent synapses following depolarization induction in hippocampal neurons (Jiang et al., 2010). It remains unclear whether such presynaptic proteins associated with exocytosis from nerve terminals are more highly expressed at synapses projecting closer to the cell body. Renger et al. (2001) revealed that functional synaptic vesicle turnover follows the localization of synapsin I with a 1–2 day delay. However, the primary factor distinguishing early-stage synaptogenesis from silent synapses remains unknown.

The NMDA receptor NR2B subunit has been reported to be replaced by NR2A during neuronal maturation, resulting in decreased exocytosis (Chavis and Westbrook, 2001). Based on these results, presynaptically silent synapses may possibly increase due to premature maturation of synapses closer to the distal cell body as neurons develop, along with an increase in the NR2A subunit. To verify this hypothesis, a qualitative determination of the expression position of the NR2A subunit is necessary. However, the regulation of expression and location of presynaptically silent synapses during development remains unclear, necessitating further research in the future.

We finally considered the difference between “real distance” and “relative distance” in the Sholl analysis. For example, in conventional Sholl analysis, the *x*-axis represents real distance (Figure 2). In other words, since dendrite extension varies even within the same group, if dendrite extension ends at, for example, 100 μm, the data for the subsequent 110–200 μm will be “0.” Consequently, the more data indicating “0,” the closer the average value to “0.” In contrast, when Sholl analysis is performed with “relative distance” (Figure 3), even if the maximum extension of dendrites ends at 100 μm, the concentric circles are divided into 5 areas. As such, even if neurons whose maximum dendrite extension ended at 200 μm and neurons whose maximum dendrite extension ended at 100 μm are mixed in the same group, the average value of area 5 will never approach “0.” Thus, the ability to obtain average values with positional information that is faithful to cell morphology is a feature and merit of Sholl analysis using “relative distance” as the *x*-axis. Still, ensuring that Sholl analysis accurately distinguishes distal from proximal synapses may be challenging. For instance, axons and dendrites can freely extend within the astrocytic dot area, and the possibility that proximal concentric rings capture distal synapses cannot be dismissed. However, this concern applies similarly to conventional Sholl analysis. Nonetheless, we trust that the findings of this study will offer insights into unraveling the mechanism of synapse development, including its unknown potential, significance, and functions.

## Data availability statement

The raw data supporting the conclusions of this article will be made available by the authors, without undue reservation.

## Ethics statement

The animal study was approved by Fukuoka University Experimental Animal Welfare Committee. The study was conducted in accordance with the local legislation and institutional requirements.

## Author contributions

OK: Data curation, Formal analysis, Investigation, Writing – original draft. KO: Data curation, Formal analysis, Investigation, Writing – original draft. KK: Validation, Writing – review & editing. TW: Validation, Writing – review & editing. SKo: Methodology, Validation, Writing – review & editing. TM: Formal analysis, Software, Validation, Writing – review & editing. SKa: Conceptualization, Data curation, Formal analysis, Funding acquisition, Investigation, Methodology, Project administration, Resources, Software, Supervision, Validation, Visualization, Writing – original draft, Writing – review & editing. KI: Validation, Writing – review & editing.
